# Role of Enhancers in Development and Diseases

**DOI:** 10.3390/epigenomes5040021

**Published:** 2021-10-04

**Authors:** Shailendra S. Maurya

**Affiliations:** Department of Pediatrics, Division of Pediatric Hematology and Oncology, Department of Developmental Biology, School of Medicine, Washington University in St. Louis, 660 South Euclid Avenue, St. Louis, MO 63110, USA; shailendrabt@gmail.com or maurya.shailendra@wustl.edu

**Keywords:** enhancer, poised, *cis*-regulatory, lineage, spatio-temporal

## Abstract

Enhancers are cis-regulatory elements containing short DNA sequences that serve as binding sites for pioneer/regulatory transcription factors, thus orchestrating the regulation of genes critical for lineage determination. The activity of enhancer elements is believed to be determined by transcription factor binding, thus determining the cell state identity during development. Precise spatio-temporal control of the transcriptome during lineage specification requires the coordinated binding of lineage-specific transcription factors to enhancers. Thus, enhancers are the primary determinants of cell identity. Numerous studies have explored the role and mechanism of enhancers during development and disease, and various basic questions related to the functions and mechanisms of enhancers have not yet been fully answered. In this review, we discuss the recently published literature regarding the roles of enhancers, which are critical for various biological processes governing development. Furthermore, we also highlight that altered enhancer landscapes provide an essential context to understand the etiologies and mechanisms behind numerous complex human diseases, providing new avenues for effective enhancer-based therapeutic interventions.

## 1. Introduction

The term “enhancer” was first coined based on studies of Simian virus 40 (SV40) when, in 1981, Banerji et al. observed for the first time that a viral DNA element from SV40 had the ability to enhance activity towards a T-antigen or β-globin reporter in mammalian cells [1]. Further research has explored endogenous sequences with similar functions in the immunoglobulin heavy chain locus. These preliminary studies have established that enhancers function as short DNA elements that trigger a gene’s transcription from a long distance in an orientation-independent manner. Preliminary studies have determined the exact mechanism of how distal regulatory elements regulate gene transcription through distal enhancers [2]. Enhancers are cis-regulatory elements that carry epigenetic information in DNA sequences through specific histone modifications [3]. Studies assessing the characteristics of enhancers have reported that they can function independently of the orientation and distance to their cognate target genes, at distances sometimes of several hundred kilobases or megabases [2,4,5]. Enhancers can be identified and characterized by various factors, including histone modifications, their transcription into non-coding RNAs, and their epigenetic features [6]. The prominent feature of enhancers is their ability to serve as a docking platform for transcription factor binding, where developmental signaling (intrinsic or extrinsic) cues are interpreted in a highly context-specific manner [7]. The signatures of these enhancers are highly cell type-specific; such cell type-specific use of the epigenetic information has demonstrated the combinatorial function of transcription factors in maintaining cell identity and lineage determination [8]. The cell type-specific enhancer pattern provides a unique cis-regulatory platform, in which transcription factors are activated by developmental cues and modify the transcriptome [9,10,11]. This model revealed that every developmental stage has a cell type-specific transcription factor, which functions by cell type-specific enhancer signatures [12]. Enhancers are actuated in a stage-specific manner, correlated with cell type-specific histone modifications. This combinatorial mechanism of transcription factor binding on cell type-specific enhancers results in the so-called enhancer signature, which serves as a readout to define enhancers in a cell type-specific manner at a global scale [13]. Precise spatial and temporal control of gene expression and the correct interpretation of the enhancer signature are crucial in the development process. Any alterations in the enhancer signature can modify the gene expression pattern and ethe capacity of the enhancer to respond to developmental signals, narrowing cell differentiation and affecting correct lineage formation. Heinz et al. (2010) have shown that lineage-determining transcription factors bind to a genomic region in a cell-specific manner. They showed the genome-wide locations of PU.1 binding patterns in macrophages, B-cells, and diffuse B-cell progenitors [14]. Similarly, Xu et al. (2012) analyzed chromatin state maps, transcription factor occupancy rates, and gene expression profiles during the development of human erythroid cells at the fetal and adult stages, and carried out a comparative analysis to determine the specific procedures of the development stage [15]. Here, it is also important to discuss the study of Choukrallah et al. (2015), who found that the enhancer landscape is dynamically reshaped in each differentiation step. Interesting changes include creating new enhancers and the closing and re-opening of the pre-existing enhancer landscape [16]. The authors also reported that the regulatory signatures of two related types of myeloid leukemia expression fusion proteins (RUNX1-ETO and RUNX1-EV1) display a distinct binding pattern and interact with different transcription factors to impact the epigenome [17]. A study has also reported that MLL-Af9 and MLL-AF4 oncofusion proteins showed distinct binding patterns in the enhancer region and targeted the RUNX1 program in 11q23 acute myeloid leukemia [18]. Enhancers are essential for normal functioning, and the loss of enhancer elements can cause abnormalities; for example, Groschel et al. (2014) showed that the removal of the distal enhancer essential for the GATA2 gene resulted in insufficient Functional GATA2 haploids, which only reduced the expression of the remaining normal alleles [19]. This review focuses on a concise and brief overview of the roles of enhancers in development and disease. We attempt to discuss how enhancers are activated and coordinated with transcription factors, as well as the roles of enhancers in mammalian development. This may comprise the first attempt to compile recently published research on development and disease, focusing on enhancers.

## 2. Features and Types of Enhancers

Enhancers have been identified in the form of various regulatory domains, primed enhancers, active enhancers, and poised enhancers. Each type of enhancer signature has specific histone modification patterns and can be easily identified by these signatures [13,20,21]. Primed enhancers can be identified by only histone H3K4 mono-methylation, while active enhancers signatures are identified by H3K4 mono-methylation and H3K27ac. Finally, the signatures of poised enhancers are marked with H3K4Me1 with H3K27me3, but not H3K27ac [13,20,21]. Active enhancers are linked to expressed genes, while poised enhancers are always associated to developmental genes, which are inactive in embryonic stem cells or precursor cells and become expressed during different differentiation stages [22]. During differentiation, the poised enhancer’s signature successfully loses the repressive H3K27me3 histone mark, acquires H3K27ac marks, and becomes active. Enhancers are, thus, subject to dynamic change functions, as an on/off switch to tune the target gene expression and changing the cell state from undifferentiated to differentiated phenotype [23]. Hence, the signatures of Poised Enhancers comprise a small set of regulatory signatures in embryonic stem cells that facilitate their timely and stage-specific function, once the correct differentiation signals become available [20]. One term also uses super-enhancers (SEs), which are described as large clusters of active enhancers with robust enrichment for binding transcriptional coactivators [24,25]. These features have also been called stretch enhancers [26], multiple enhancers [27], and enhancer clusters [28], which are similar but not identical between studies (although many of these features overlap). SEs regulate master regulators of pluripotency, such as OCT4, SOX2, and NANOG [24]. It has been reported that the SE signature is often enriched near the oncogene in tumor cells, while an enrichment GWAS has identified SNPs normally associated with several common diseases [29,30]. 

## 3. Enhancers and Lineage Determination during Development

**Figure 1:**
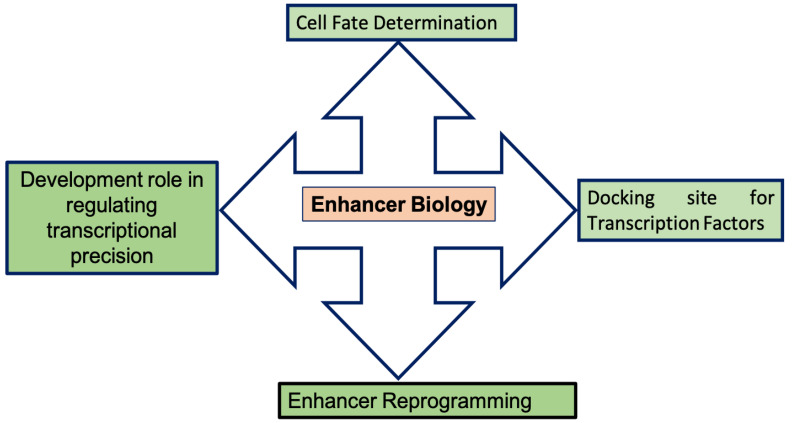
Schematic diagram representing enhancer biology.

Many studies have established the idea that the enhancer signature is intricately orchestrated, in a stage-specific pattern, by several proteins complexes during development [31]. Some enhancer signatures are established early during development in precursor cells, and are modified and activated as cells differentiate in terminal steps along specific lineages [32]. All enhancer marks serve different purposes; for example, poised enhancers serve as signatures for future gene expression [2]. In contrast, active enhancer signatures play a functional role in the current transcriptional state [33]. The chromatin state is mostly invariant across different tissues, whereas histone signature patterns at the enhancer level are highly tissue-specific [34]. Mammals, with about 200 specialized cell types, all have different transcriptional outcomes, reflecting the unique coding pattern and regulatory elements during development [34,35,36]. Indeed, the enhancer repertoire active in a particular lineage, as identified by chromatin marks and transcriptional regulators, represents only a small subset of all genomic regulatory domains [33,37,38]. Studies have suggested that the enhancer repertoire is a pre-established landscape, formed and imposed by the lineage, determining TFs that maintain cell identity. All transcriptional regulation and functional outcomes occur within the differentiated cell under this pre-established enhancer landscape [10]. The study also established that enhancers playing a developmental role are evolutionarily conserved sequences [39,40]. Thus, the pre-established enhancer landscape has a crucial role in lineage determination. Any disturbance in the enhancer landscape affects the lineage, determining the potential of cells. This concept suggests that any external cues that trigger the transitory response cannot functionally change the repertoire of genomic regulatory domains, but act on the pre-established epigenomic landscape. This response is a buffer system that ensures cell identity maintenance, despite the changing environment [10]. A schematic diagram explaining enhancer biology is shown in Figure 1. We also summarize the related studies in Table 1, which have investigated the roles of enhancers in development through different model systems, including evidence in support of early enhancer establishment reported in B-cell and macrophage specification [14,16], T-cell development [41], early hematopoiesis [42], and the commitment of multipotent endoderm cells to liver and pancreas cell fates [43]. Wang et al. (2015) have also inferred the role of the poised enhancer landscape in endoderm development, as well as established a functional link between the gain of poised enhancer chromatin state and the temporal acquisition of competence during developmental progression [44]. Dynamic and coordinated epigenetic regulation has also demonstrated chromatin transition during cardiac lineage commitment [45]. Stage-specific enhancers are synergistically activated in a genome-wide manner during cardiac development by cardiac reprogramming factors [46]. The combinatorial action of pioneer factor and super-enhancer dynamics has also been reported in stem cell plasticity and lineage choice [47]. Further, Enhancer priming by histone methyl-transferase has also been demonstrated to control cell fate transition [48]. The master regulator ‘Scl’ has been reported to bind to pre-established primed enhancer signatures in the mesoderm, as well as regulating hematopoietic and cardiac fate divergence at terminal differentiation steps. Scl uses the pre-established epigenetic landscape during the specification of lineage choices [49]. The pioneer factor FOXA2 is required for enhancer priming during HPSC differentiation in pancreatic lineages [50]. It has also been shown that ERK directly regulates enhancer priming in lineage choice [51]. Developmental stage-specific enhancers drive lineages and control gene expression programs during hematopoiesis [15,52]. Rubin et al. (2017) have reported that lineage-specific dynamic and pre-established enhancer–promoter contacts cooperate in terminal differentiation [53]. Recently, Maurya et al. (2021) [54] have shown that the loss of KMT2C in HPSCs cells significantly reprograms the enhancer landscapes in HPSCs cells, leading to the loss of Hemogenic endothelium during in vitro hematopoietic differentiation. Further, it has also been reported that the deletion of KDM6A in HPSCs cells significantly reprograms the Bivalent chromatin in HPSCs cells, suggesting perturbed development at the terminal developmental steps in particular lineages [55].

## 4. Enhancer–Promoter Interaction Is the Core Key to Regulating Gene Expression

The precise cell type-specific expression of genes often needs additional cis-regulatory elements, which are physically distanced from the promoter regions. These distal cis-regulatory components harbor TFs that are highly cell type-specific and are expressed in the presence of external developmental cues, such as stage-specific signals during differentiation or proliferation. These regulatory elements set complex gene expression patterns at different developmental stages and time points, by combining other external developmental cues [30]. The enhancer element interacts with a promoter, independent of direction and distance, to activate transcription. The most elegant choreography of the biology of these distal regulatory elements is how enhancers identify their cognate promoters and initiate transcription, in addition to the underlying mechanisms that regulate these preinitiation assemblies. It has been shown that enhancers regulate transcription by a looping mechanism between the enhancers and their cognate promoters. Therefore, a more direct physical contact of cis-regulatory elements is established within the nucleus [60]. This mechanism, underlying the crosstalk between enhancers and promoters, has been considered to explore how long-range interactions form chromatin loops. A strategic enhancer is used to interact with its cognate promoter during the initiation of transcription [60]. This entire cyclic strategy of remote regulatory elements has also been observed for the cytokine locus of t-helper type 2 (TH2) cells [61]. The ability of cis-regulatory elements to communicate with promoters is not restricted to a gene position situated at only cis positions on the same chromosome. Reports are also available which have shown that the olfactory H enhancer elements can communicate with multiple olfactory genes with different chromosomes in epithelial tissues, where these genes are normally expressed [4]. These studies suggest that proper gene expression requires the proper establishment of enhancer–promoter interactions. Enhancers serve as docking sites for TFs, and the activity of enhancers is mostly based on the binding of these TFs [11]. More than a thousand TFs encode the human genome. Open chromatin DNA regions have short (20–30 bp) DNA sequences normally occupied with TFs within the enhancer sequences, known as DNA recognition motifs, which are also characterized by low nucleosome occupancy. Some TFs are cell type-/lineage-specific, while some TFs are bound across different cell types [62,63]. For example, GATA1 is known explicitly for erythroid differentiation, which is required in hematopoietic differentiation, and PU.1 is important for B-cell specification within a hematopoietic lineage [64,65]. Furthermore, enhancers can recruit several additional factors to maintain fine-tuned targeted spatio-temporal expression of genes. The functional roles of epigenetic writers and erasers are also important for enhancers, leading to specific epigenetic modifications in the enhancers. For example, COMPASS complexes (KMT2A, KMT2B, KMT2C, and KMT2D) are histone methyl-transferases that bind cis-regulatory elements mediating the methyl marks on histone [66,67]. These chromatin remodelers and TFs can affect the nucleosome dynamics, thus regulating the enhancer regions. Different TFs show cooperative binding in nucleosome-embedded motifs while, alone, the TFs can bind weakly. TFs can also cooperate with chromatin remodeling factors to accelerate the auxiliary loading mechanism. Although the initial association of a TF with its TFBSc can recruit chromatin modulators near the closed chromatin, it initiates the position and promotes the later binding of secondary TFs and accelerates the positive feedback mechanism [68,69,70]. Several studies have shown that the enhancer epigenetic landscape is intricately orchestrated in a set pattern by several regulatory protein complexes during development [32].

## 5. Role of Enhancers in Disease Development

The genome-wide sequencing approach has revealed that enhancers are prime targets for genetic or epigenetic alterations that lead to carcinogenesis [5]. The main characteristics of the enhancer signature remain constant for every type of cell (e.g., normal vs. cancer/tumor cells). The difference is that the function of the enhancer signatures differs between normal and cancer cell types; that is, the enhancer’s functional output in tumor/cancer cells differs from that in normal cells [71]. Careful examination of enhancer signatures between normal cells and their counterpart in cancer cells has revealed that cancer cells tend to lose enhancers at positions near cell fate-specifying genes and gain enhancer signatures near growth-associated genes [72,73,74]. Furthermore, it has again been confirmed, after comparing normal colon epithelial crypts and colon cancer lines, that thousands of differentially enriched primed enhancer marks (H3K4Me1) were enriched in cancerous cells known as variant enhancer loci (Veli), and these gained mono-methylation sites were not found in normal counterpart cells. In contrast, the lost sites were relatively specific to crypt cells, indicating that the colon cancer cells acquired a more differentiated cell state [73]. In the post-GWAS era, there is a lot of conceivable evidence that cancer predisposition genomic variants present in the non-coding genomic region lie within these distal regulatory elements [75,76]. This finding is based on the overlap between SNPs associated with diseases and genomic signatures, revealing that SNPs associated with risk phenotypes are frequently enriched in expression quantitative traits and open chromatin regions [60,77]. In cancer cells, risk variants lead to the dysregulation of enhancers, disrupting the fine-tuned target expression of their associated genes and producing a pathological state which leads to abnormal growth. For example, pancreatic-specific differential open regions enriched for non-coding variants (SNPs) have been linked to pancreatic disorders [78]. Correspondingly, monocyte-/macrophage-specific enhancer elements enriched with SNPs associated with ulcerative colitis, celiac disease, Crohn’s disease, or systemic lupus erythematosus have been reported [79]. Studies are also available in which single base-pair point mutations in the distal enhancer of SCNA and PTF1A have been shown to cause sporadic Parkinson’s disease and pancreatic agenesis [80,81]. Altered super-enhancer activity has also been reported in many complex human diseases, such as Alzheimer’s, Type 1 diabetes, and autoimmune disorders [25,28,82,83]. Furthermore, lost super-enhancers and enhancers acquired somatically have been reported to be associated with numerous cancers [25,82]. Meanwhile, changes in histone writers, erasers, and altered activity of DNA modifiers such as methyl and acetyltransferases also reshape the chromatin landscape, which finally leads to abnormal growth and development. Based on known enhancer signatures, several studies (Table 2) have explored the alterations in enhancer activity and histone modification patterns which may be correlated with disease development. Evidence for enhancer alterations in cancer, coming from various studies, is summarized in Table 2.

## 6. Enhancer Reprogramming

The role of enhancer reprogramming is an emerging area in developmental biology and cancer research. The emerging role of enhancers in fate determination has now been well-established. In cancer research, enhancers have been shown to acclimatize cancer cells to environmental changes encountered during cancer progression and development [127]. Recent findings have highlighted that enhancer reprogramming plays a crucial role in carcinogenesis and metastasis formation, thus playing a role in establishing malignant heterogeneity [127,128]. A cancer genomics study has identified that recurrent genetic mutations mostly occur on genes coding for epigenetic modifiers [129]. In addition to the dysregulation of these trans-epigenetic regulators, genome-wide sequencing of the non-coding genome has evidenced the frequent alterations of cis-regulatory elements, such as enhancers and insulators [71]. Considering that enhancer activity modulation plays a significant role in maintaining and controlling the cell identity and cell adaptation to environmental changes, it is easily conceivable that genetic alternations affecting the epigenetic modifiers and cis-regulatory machinery may alter enhancer activity, thus affecting cell fate determination [130]. Specifically, recent studies have suggested that enhancer function reprogramming could represent a hallmark of carcinogenesis, as it contributes to the deregulated expression of epigenetic modifiers, leading to abnormal cell growth. In this respect, oncogenic enhancer reprogramming, considered as cancer-related alterations, may cause aberrant oncogenic development, leading to altered transcription outputs, thus promoting carcinogenesis. Recent conceptually interesting studies have suggested that carcinogenesis involves transforming the cell to a more primitive stage (that is, a developmentally manipulable differentiation state) driven by major changes in the epigenomic landscape [131]. This work highlighted several thought-provoking questions: First, while enhancers are indispensable for specifying transcriptional outputs that drive cell fate determination, it is interesting that a huge shift of enhancer landscape use is particularly critical for PDA metastasis [131]. It has recently been shown that genetic alterations could induce genetic priming in the cell of origin, imposing the primed cell’s phenotype [132]. This response, thus, indicates a new function for genetic insult. This new transformation mechanism reveals that the first aberrant oncogenic hit imposes a cell differentiation program in cancer-initiating cells which is responsible for the tumor cell phenotype at terminal differentiation steps [132]. It is not necessary that the first oncogenic phenotypes for tumor initiation will be responsible for the altered differentiation program. It has been observed that the oncogene is not at all required in the terminal steps of transformation. This suggests that initial hits are not good targets for therapies, as they do not play an essential role in tumor development [133]. Specific epigenomic profiles can distinguish between different lineages in the hematopoietic differentiation system, as well as in leukemic cells [134]. This epigenomic reprogramming has been observed in some other animal cancer models which resemble human cancer. A recent study by Adelman et al. (2019) showed that age-associated epigenetic reprogramming may form a predisposing condition for the development of age-related AML [135].

## 7. Concluding Remarks

This review discussed how enhancer signatures function as a critical platform, acting as a receiver for extrinsic and intrinsic developmental signals, as well as conveying the context-specific manner during lineage determination. However, research into the functional annotation of enhancer readout in cancer and the development-specific roles of enhancer biology are still in an initial phase, and several unanswered questions remain poorly explored. Enhancer signatures can potentially be used as signature biomarkers for disease-specific phenotype identification and cancer detection. Although, at present, limited resources are available which correlate enhancer signatures with clinical outcomes, future studies are expected to shed more light on the regulatory mechanisms that modify the chromatin landscape patterns at distal regulatory sequences during the cell’s different stages of development and cell differentiation. Multiomics studies, involving genomics, epigenomics, deep global proteomics, single-cell heterogeneity, and single cell epigenomics in different developmental systems, will provide more concrete information on how histone pre-patterns and chromatin modifiers regulate the chromatin landscape under specific enhancer signatures during the developmental process, as well as their impact on phenotypes. Hopefully, new emerging technologies and genome editing tools will uncover novel insights into the roles of enhancer signatures in regulating developmental processes. Future studies should focus on exploring the roles of enhancers and how cell type-specific gene expression can be pre-established and maintained throughout the terminal steps.

## Figures and Tables

**Table 1:** Studies investigating the roles of enhancers in development.

Number	Title	Author	Reference
1	Highly conserved non-coding sequences are associated with vertebrate development	Woolfe et al., 2005	[39]
2	Simple Combinations of Lineage-Determining Transcription Factors Prime cis-Regulatory Elements Required for Macrophage and B Cell identities	Heinz et al., 2010	[14]
3	Transcriptional enhancers in animal development and evolution	Levine Mike, 2010	[40]
4	A unique chromatin signature uncovers early development enhancers in humans	Rada-Iglesias et al., 2011	[20]
5	Multilineage Priming of Enhancer Repertoires Precedes Commitment to the B and Myeloid Cell Lineages in Hematopoietic Progenitors	Mercer et al., 2011	[52]
6	Chromatin “Prepattern” and Histone Modifiers in a Fate Choice for Liver and Pancreas	Xu et al., 2011	[43]
7	Foxp3 Exploits a Pre-Existent Enhancer Landscapes for Regulatory T Cell Lineages Specification	Samstein et al., 2012	[41]
8	A Temporal Chromatin Signature in Human Embryonic Stem Cells Identifies Regulators of Cardiac Development	Paige et al., 2012	[46]
9	Combinatorial Assembly of Development Stage-Specific Enhancers Controls Gene Expression Programs during Human Erythropoiesis	Xu, J. et al., 2012	[15]
10	Dynamic and Coordinated Epigenetic Regulation of Developmental Transitions in the Cardiac Lineage	Wamstad et al., 2012	[45]
11	Enhancers as information integration hubs in development: lesson from genomics	Buecker & Wysocka, 2012	[6]
12	Developmental Fate and Cellular Maturity Encoded in Human Regulatory DNA Landscapes	Stergachis et al., 2013	[56]
13	Latent Enhancers Activated by Stimulation in Differentiated Cells	Ostuni et al., 2013	[10]
14	Epigenetic Priming of Enhancers Predicts Developmental Competence of hESC-Derived Endodermal Lineage Intermediates	Wang et al., 2015	[44]
15	Scl binds to primed enhancers in mesoderm to regulate hematopoietic and cardiac fate divergence	Org et al., 2015	[49]
16	Pioneer factors govern super-enhancer dynamics in stem cell plasticity and lineage choice	Rc et al., 2015	[47]
17	Early enhancer establishment and regulatory locus complexity shape transcriptional programs in hematopoietic differentiation	Gonzalez et al., 2015	[42]
18	Enhancer repertoires are reshaped independently of early priming and heterochromatin dynamics during B cell differentiation	Choukrallah et al., 2015	[16]
19	Lineage-Specific Genome Architecture Links Enhancer and Non-coding Disease Variants to Target Gene Promoters	Javierre et al., 2016	[57]
20	Enhancer priming by H3K4 methyltransferase MLL4 controls cell fate transition	Wang et al., 2016	[48]
21	Ever Changing landscape: transcriptional enhancers in development and evolution	Long et al., 2016	[58]
22	Lineage-specific dynamic and pre-established enhancer-promoter contacts cooperate in terminal differentiation	Rubin, J.A. et al., 2017	[53]
23	Dynamic lineage priming is driven via direct enhancer regulation by ERK	Hamilton et al., 2019	[51]
24	FOXA2 is Required for Enhancer Priming during Pancreatic Differentiation	Lee et al., 2019	[50]
25	Cardiac Reprogramming Factors Synergistically Activate Genome-wide Cardiogenic Stage-Specific Enhancers	Hashimoto et al., 2019	[59]

**Table 2:** Studies investigating the roles of Enhancers in disease development.

Number	Title	Author	Reference
1	Translocation of the c-myc gene into the immunoglobulin heavy chain locus in human Burkitt lymphoma and murine plasmacytoma cells	Taub et al., 1982	[84]
2	A long-range Shh enhancer regulates expression in the developing limb and fin and is associated with preaxial polydactyly	Lettice, L.A. et al., 2003	[85]
3	Genomic deletion of a long-range bone enhancer misregulates in Van Buchem disease	Loots, G.G. et al., 2005	[86]
4	A common sex dependent mutations in a RET enhancer underlies Hirschsprung disease risk	Emison, E.S. et al., 2005	[87]
5	Disruption of an AP2-alpha binding site in an IRF6 enhancer is associated with cleft lip	Rahimov et al., 2008	[88]
6	Functional enhancers at the gene-poor 8q24 cancer linked locus	Jia, L. et al., 2009	[89]
7	The 8q24 cancer risk variant rs6983267 shows long-range interaction with MYC in colorectal cancer	Pomeratz, M.M. et al., 2009	[90]
8	The common colorectal cancer predisposition SNP rs6983267 at chromosome 8q24 confers potential to enhanced WNT signaling	Tuupanen, S. et al., 2009	[91]
9	Long-range enhancers on 8q24 regulate c-Myc	Sotelo et al., 2010	[92]
10	An 8q24 gene desert variant associated with prostate cancer risk confers differential in vivo activity to a MYC enhancer.	Wasserman, N.F. et al., 2010	[93]
11	Enhancer-adoption as a mechanism of human developmental disease	Lettice, L.A. et al., 2011	[94]
12	Systematic localization of common disease associated variation in regulatory DNA	Maurano et al., 2012	[76]
13	Epigenomic enhancer profiling defines a signature of colon cancer	Akhtar-Zaidi, B. et al., 2012	[73]
14	Mice lacking a Myc enhancer that includes human SNP rs6983267 are resistant to intestinal tumors	Sur et al., 2012	[95]
15	A novel 13 base pair insertion in the sonic hedgehog ZRS limb enhancer (LMBR1) causes preaxial polydactyly with triphalangeal thumb	Laurell, T. et al., 2012	[96]
16	Regulatory variation in a TBX5 enhancer leads to isolated congenital heart diseases	Smemo, S. et al., 2012	[97]
17	DNA methylation of transcriptional enhancers and cancer predisposition	Aran and Hallman et al., 2013	[98]
18	Discovery and characterization of super-enhancer associated dependencies in diffuse large B cell lymphoma	Chapuy, B. et al., 2013	[99]
19	Chromatin stretch enhancer states drive cell specific gene regulation and harbor human disease risk variants	Parker, S.C. et al., 2013	[26]
20	Role of SWI/SNF in acute leukemia maintenance and enhancer-mediated Myc regulation	Shi, J. et al., 2013	[100]
21	Selective inhibition of tumor oncogenes by disruption of super-enhancers	Loven, J. et al., 2013	[29]
22	An erythroid enhancer of BCL11A subject to genetic variation determines fetal hemoglobin level	Bauer, D.E. et al., 2013	[101]
23	Disruption of autoregulatory feedback by a mutation in a remote, ultraconserved PAX6 enhancer causes aniridia	Bhatia, S. et al., 2013	[102]
24	Genome-wide analysis of noncoding regulatory mutations in cancer	Weinhold, N. et al., 2014	[103]
25	A NOTCH driven MYC enhancer promotes T cell development, transformation and acute lymphoblastic leukemia	Herranz, D. et al., 2014	[104]
26	Combinatorial effects of multiple enhancer variants linkage disequilibrium dictate levels of gene expression to confer susceptibility to common traits	Corradin, O. et al., 2014	[27]
27	Enhancer hijacking activates GFI1 family oncogenes in medulloblastoma	Northcott, P.A. et al., 2014	[105]
28	Microduplications encompassing the sonic hedgehog limb enhancer ZRS are associated with Hass-type polysyndactyly and Laurin Sandrow syndrome	Lohan, S. et al., 2014	[106]
29	Epigenomic analysis of primary human T cells reveals enhancers associated with TH2 memory cell differentiation and asthma susceptibility	Seumois, G. et al., 2014	[107]
30	Oncogenic regulation. An oncogenic Super enhancer formed through somatic mutation of a noncoding intergenic element.	Mansour, M.R. et al., 2014	[108]
31	A Sox2 distal enhancer cluster regulates embryonic stem cell differentiation potential	Zhou, H.Y. et al., 2014	[109]
32	Multiple functional risk variants in a SMAD7 enhancer implicate a colorectal cancer risk haplotype	Fortini, B.L. et al., 2014	[110]
33	A remote GATA2 hematopoietic enhancer drives leukemogenesis in inv(3) (q21;q26) by activating EVI1 expression	Yamazaki et al., 2014	[111]
34	Long range enhancer activity determines Myc sensitivity to Notch inhibitors in T cell leukemia	Yashiro-Ohtani et al., 2014	[112]
35	Recessive mutations in a distal PTF1A enhancer cause isolated pancreatic agenesis	Weedon et al., 2014	[81]
36	A single oncogenic enhancer rearrangement causes concomitant EV1 and GATA2 deregulation in leukemia	Groschel et al., 2014	[19]
37	Genetic predisposition to neuroblastoma mediated by a LMO1 super enhancer polymorphisms	Oldridge, D.A. et al., 2015	[113]
38	7q21.3 Deletion involving enhancer sequences within the gene DYNC1I1 presents with intellectual disability and split hand-split foot malformation with decreased penetrance	Delgado, S. and Velinov, M., 2015	[114]
39	A large genomic deletion leads to enhancer adoption by the lamin B1 gene: a second path to autosomal dominant adult-onset demyelinating leukodystrophy (ADLD)	Giorgio, E. et al., 2015	[115]
40	Multiple functional variants in long-range enhancer contribute to the risk of SNP rs965513 in thyroid cancer	He, H. et al., 2015	[116]
41	Loss of TET2 in hematopoietic cells leads to DNA hypermethylation of active enhancers and induction of leukemogenesis	Rasmussen et al., 2015	[117]
42	The Transcriptional cofactor TRIM33 prevents apoptosis B lymphoblastic leukemia by deactivating a single enhancer	Wang et al., 2015	[118]
43	Super-enhancers delineate disease-associated regulatory nodes in T cells	Vahedi et al., 2015	[83]
44	Identification of focally amplified lineage-specific super-enhancers in human epithelial cancer	Zhang, X. et al., 2016	[119]
45	Role of non-coding sequence variants in cancer	Khurana, E. et al., 2016	[120]
46	Ever-changing landscapes: transcriptional enhancers in development and evolution	Long, H.K. et al., 2016	[58]
47	Genetic Predisposition to Chronic Lymphocytic Leukemia is mediated by a BMF Super-Enhancer Polymorphisms	Kandaswamy et al., 2016	[121]
48	DNMT3A Loss drives Enhancer Hypomethylation in FLT3-ITD-Associated Leukemias	Yang et al., 2016	[122]
49	Epigenomic profiling of primary gastric adenocarcinoma reveals super-enhancer heterogeneity	Ooi et al., 2016	[82]
50	Parkinson associated risk variants in distal enhancers of a-syncuclein modulates target gene expression	Soldner et al., 2016	[80]
51	Hotspots of aberrant enhancer activity punctuate the colorectal cancer epigenome	Cohen, A.J. et al., 2017	[30]
52	Composition and dosage of a multipartite enhancer cluster control developmental expression of Ihh (Indian hedgehog)	Will, A.J. et al., 2017	[123]
53	Superenhancer Analysis Defines Novel Epigenomic Subtypes of Non-APL AML, including an RARaalpha Dependency Targetable by SY-1425, a Potent and Selective RARalpha Agonist	MCKeown, M.R. et al., 2017	[124]
54	Enhancer profiling identifies critical cancer genes and characterize cell identity in adult T-cell leukemia	Wong et al., 2017	[125]
55	APOBEC signature mutation generate an oncogenic enhancer that drives LMO1 expression in T-ALL Leukemia	Li et al., 2017	[126]
